# Adenosine: a partially discovered medicinal agent

**DOI:** 10.1186/s43094-021-00353-w

**Published:** 2021-10-21

**Authors:** Rohit Batra, Vinay Jain, Pankaj Sharma

**Affiliations:** 1 Department of Pharmacology, ShriRam College Pharmacy, Banmore, Morena, M.P 476444 India; 2 Department of Pharmacognosy, ShriRam College Pharmacy, Banmore, Morena, M.P 476444 India; 3 Department of Pharmaceutics, ShriRam College Pharmacy, Banmore, Morena, M.P 476444 India

**Keywords:** Adenosine, G protein-coupled receptor (GPCR), Bone remodelling, Myocardial perfusion imaging, Inflammation, Pathogenesis

## Abstract

**Background:**

A plethora of chemicals exists in human body which can alter physiology in one way or other. Scientists have always been astounded by such abilities of chemicals but as the technology advances, even the chemical which was once expected to be well known changes its status to not really well known. Adenosine is one of the chemicals which is in consonance with the aforementioned statements, although previous articles have covered vast information on role of adenosine in cardiovascular physiology, bacterial pathophysiology and inflammatory diseases. In this review we have discussed adenosine and its congeners as potential promising agents in the treatment of Huntington’s disease, post-traumatic stress disorder, erectile dysfunction, viral infections (SARS-CoV) and anxiety.

**Main text:**

Adenosine is a unique metabolite of ATP; which serves in signalling as well. It is made up of adenine (a nitrogenous base) and ribo-furanose (pentose) sugar linked by β-N9-glycosidic bond. Adenosine on two successive phosphorylation forms ATP (Adenosine Triphosphate) which is involved in several active processes of cell. It is also one of the building blocks (nucleotides) involved in DNA (Deoxy-ribonucleic Acid) and RNA (Ribonucleic Acid) synthesis. It is also a component of an enzyme called S-adenosyl-L-methionine (SAM) and cyano-cobalamin (vitamin B-12). Adenosine acts by binding to G protein-coupled receptor (GPCR: A1, A2A, A2B and A3) carries out various responses some of which are anti-platelet function, hyperaemic response, bone remodelling, involvement in penile erection and suppression of inflammation. On the other hand, certain microorganisms belonging to genus *Candida, Staphylococcus and Bacillus* utilize adenosine in order to escape host immune response (phagocytic clearance). These microbes evade host immune response by synthesizing and releasing adenosine (with the help of an enzyme: adenosine synthase-A), at the site of infection.

**Conclusion:**

With the recent advancement in attribution of adenosine in physiology and pathological states, adenosine and its congeners are being looked forward to bringing a revolution in treatment of inflammation, viral infections, psychiatric and neurodegenerative disorders.

## Background

A plethora of chemical exists which can modify human physiology in multiple ways. Scientists have always been astounded by the way in which the biochemicals modify these physiology. However, with the advent of new technologies, as more and more knowledge, even the chemical which was expected to be well known often changed its status to not really well known.

This paper is about one such biochemical: adenosine. Adenosine is a metabolite, a neurotransmitter, a signalling agent which was once known to modify only cardiovascular physiology but today, it has been found to be multifaceted chemical which can alter physiology in innumerable ways. Adenosine and its congers today have served as a prototype which on more studies are expected to turn out as promising agent in treatment of several ailments and diseases.

Adenosine is one of the life sustaining chemical moieties present in cell [1]. Adenosine has nitrogenous base: Adenine, which is linked to ribose sugar (ribo-furanose, a five carbon containing sugar) via β-N9-glycosidic linkage. Adenosine is one of the essential nucleotides required in biosynthesis and duplication/replication of DNA and RNA [2–6]. ATP, the energy currency of cell, is formed from adenosine after series of steps. Hence, a majority of active process in cell will retard in the absence of adenosine making it an indispensable chemical moiety in cell [7, 8].

**Fig. 1:**
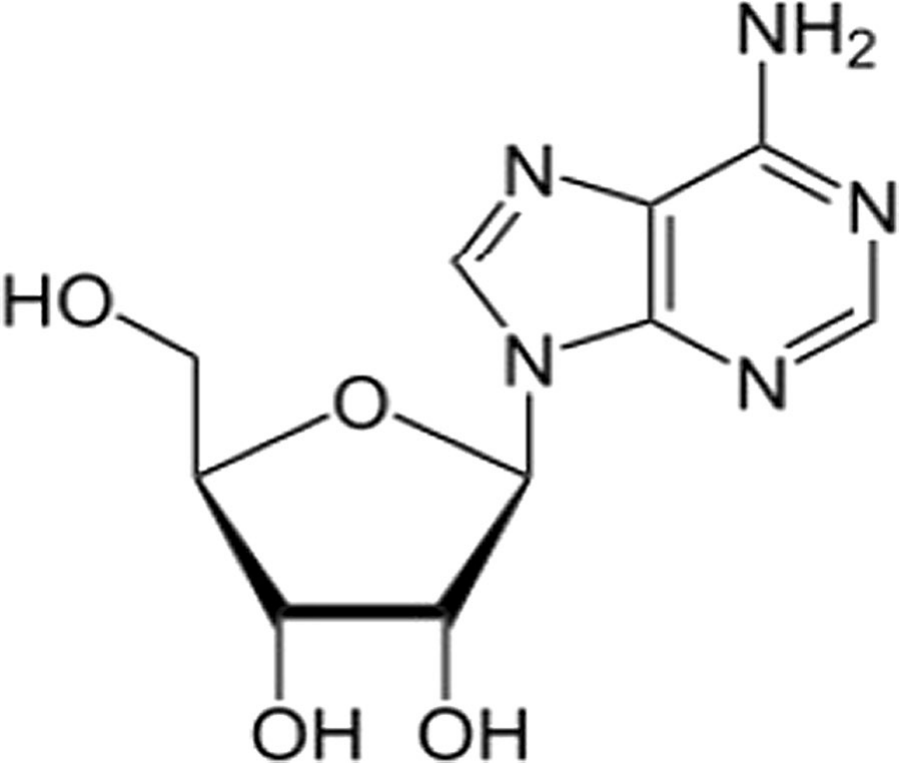
Structure of adenosine

Apart from all these functions, adenosine is also a component of an enzyme called S-adenosyl-L-methionine (SAM) and cyano-cobalamin (vitamin B-12) (Fig. 1).

## Main text

### Metabolism of adenosine

#### External or extracellular metabolism

- Adenosine deaminase enzyme is responsible for biotransformation of adenosine to inosine.
- Extracellular activity of adenosine is also brought to an end by ENT (Equilibrative Nucleoside Transporters). ENTs are responsible for intracellular influx/transport of adenosine

#### Internal or intracellular/cytosolic metabolism

- Adenosine deaminase: converts adenosine to inosine.
- S-adenosyl-homocysteine hydrolase (SAHH): converts adenosine to adenosyl homocysteine
- Adenosine kinase: converts adenosine to adenosine monophosphate by phosphorylation. This phosphorylation maintains cytosolic concentration of adenine nucleosides/nucleotides.

During cellular injury or hypoxia/ischemia, ATP is broken down to ADP by CD-39 [9]. ADP is further broken down into adenosine using CD-73. Adenosine binds to its GPCR and performs physiological–pathological actions. Adenosine is carried inside cell using transmembrane protein called equilibrative nucleoside transporters (ENTs). Adenosine is biotransformed either into inosine or S-(5′-Adenosyl)-L-homocysteine (AdoHcy) using adenosine deaminase (ADA) and S-adenosyl-L-homocysteine hydrolase (SAHH), respectively. ATP formed inside cell can migrate out of cell using connexin [10–18].

### How adenosine acts? (receptors and traducer pathways)

Adenosine acts by binding to metabotropic G protein-coupled receptors (GPCR). It activates both Gs and Gi subtypes of GPCR. A detailed explanation is given in Table 1.

**Table 1:** Various adenosine receptors, their transducer pathways, location and actions

Adenosine receptor subtype	Transducer mechanism	Pharmacological actions
A1	Linked to Gi (subtype of heterotrimeric GTP binding protein). Decreases cAMP, increases potassium ion efflux and decreased calcium ion influx	Depressing effect on myocardium (Negative Dromotropy and Negative Chronotropy). Inhibits atrioventricular conduction, prolongs refractory period
A2A and A2B	Linked to Gs (subtype of heterotrimeric GTP binding protein). Increases cAMP (Cyclic Adenosine Monophosphate)	Causes vasodilation of myocardial microvasculature. Causes constriction of hepatic vein, renal vein and afferent arterioles of spleen. Causes inhibition of platelet activity
A3	Linked to Gi	Involved in ischemic preconditioning of myocardium. Involved in promoting inflammation by attracting white blood cells; specifically neutrophils (chemo-kinesis)

ATP or adenosine triphosphate is stored inside the vesicle in presynaptic neuron. During action potential, vesicle gets fuse with presynaptic membrane of presynaptic neuron and glutamate and ATP are released. ATP is converted to adenosine by ectonucleotidase (EctoN). The released adenosine can bind to presynaptic A2A and A1 inhibitory receptor or may bind to excitatory A2A post-synaptic receptor. Adenosine is taken up by equilibrative nucleoside transporters (ENT) inside cell where it is phosphorylated by kinases to ADP and ATP. A2A adenosine receptors on blood vessels exert vasodilatory effect [19–27].

### Pharmacological and pathological roles of adenosine

#### Adenosine in hyperaemic response

Myocardium of human heart is supplied oxygen by coronary micro-circulation. In response to hypoxia/ischemia, coronary perfusion multiplies (almost 5 times the base value): a phenomenon known as hyperaemic response. Adenosine is also one of the factors which are involved in hyperaemic response because of its ability to cause vasodilation in coronary microvasculature. Cytosolic level of adenosine increases at the time of ischemia since there is a misalliance between its use and ATP synthesis (in the absence of oxygen; synthesis of ATP is retarded) [28, 29].

#### Adenosine in bone remodelling

Adenosine plays a significant role in bone reabsorption and remodelling. By activating A1 receptor, development of osteoclast from monocytic precursor is augmented. Activation of A2 receptor on the contrary inhibits differentiation of osteoclast in bones [30–35].

#### Adenosine as anti-platelet agent

Adenosine and one of its analogues 2-chloro adenosine have been indicated as anti-platelet agents. They act by activating two subtypes of adenosine receptor, A2A and A2B. This agonism results in increased levels of cAMP inside thrombocytes/platelets resulting in anti-platelet activity.

Although standard anti-platelet regimen includes a combination therapy with at least two anti-platelet drugs (e.g. aspirin and clopidogrel) yet patients (especially of type II Diabetes Mellitus) express resistance to these drugs after a course of time. Adenosine receptor agonist has shown an ability to curb this resistance [36–38]

**Fig. 2:**
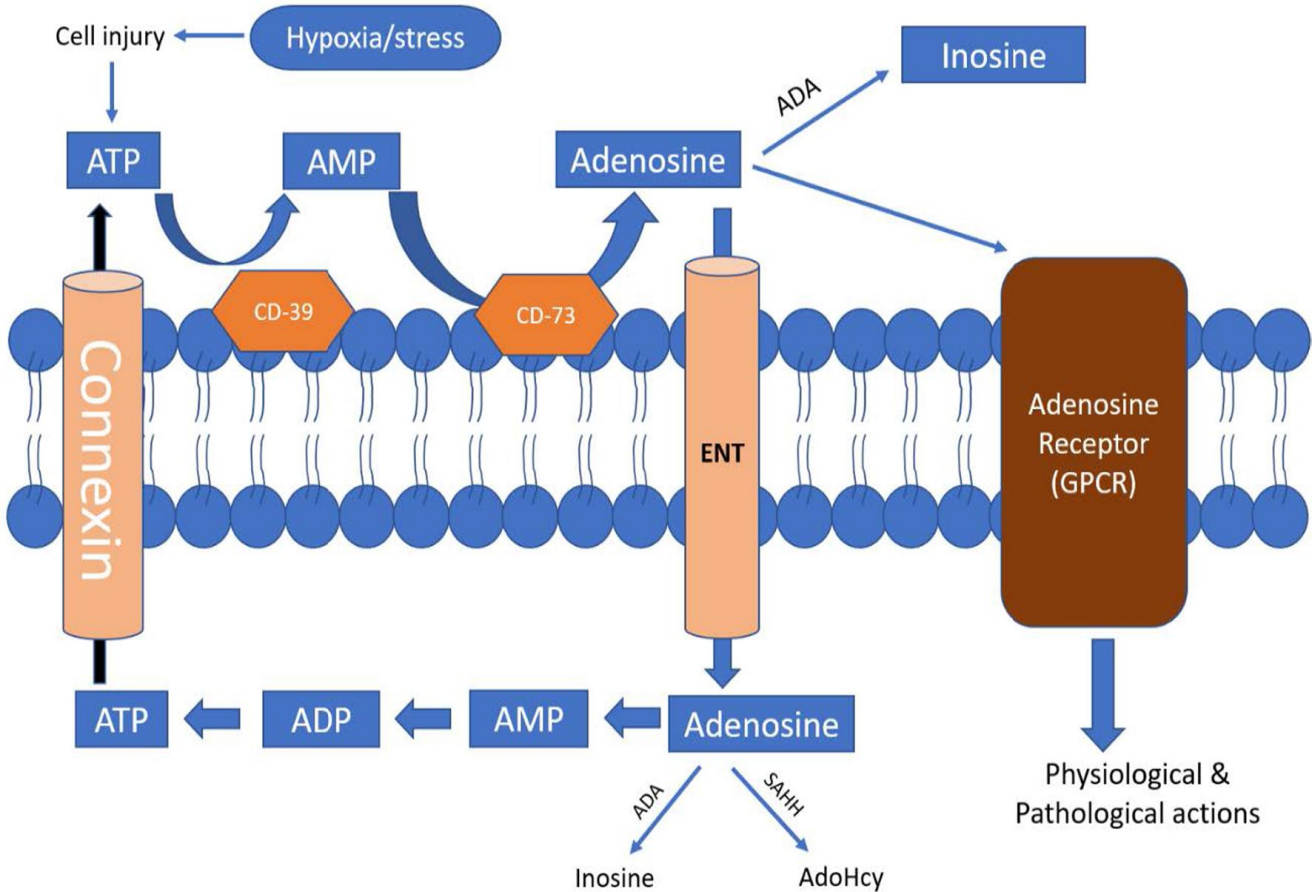
Catabolism of ATP and formation of adenosine

**Fig. 3:**
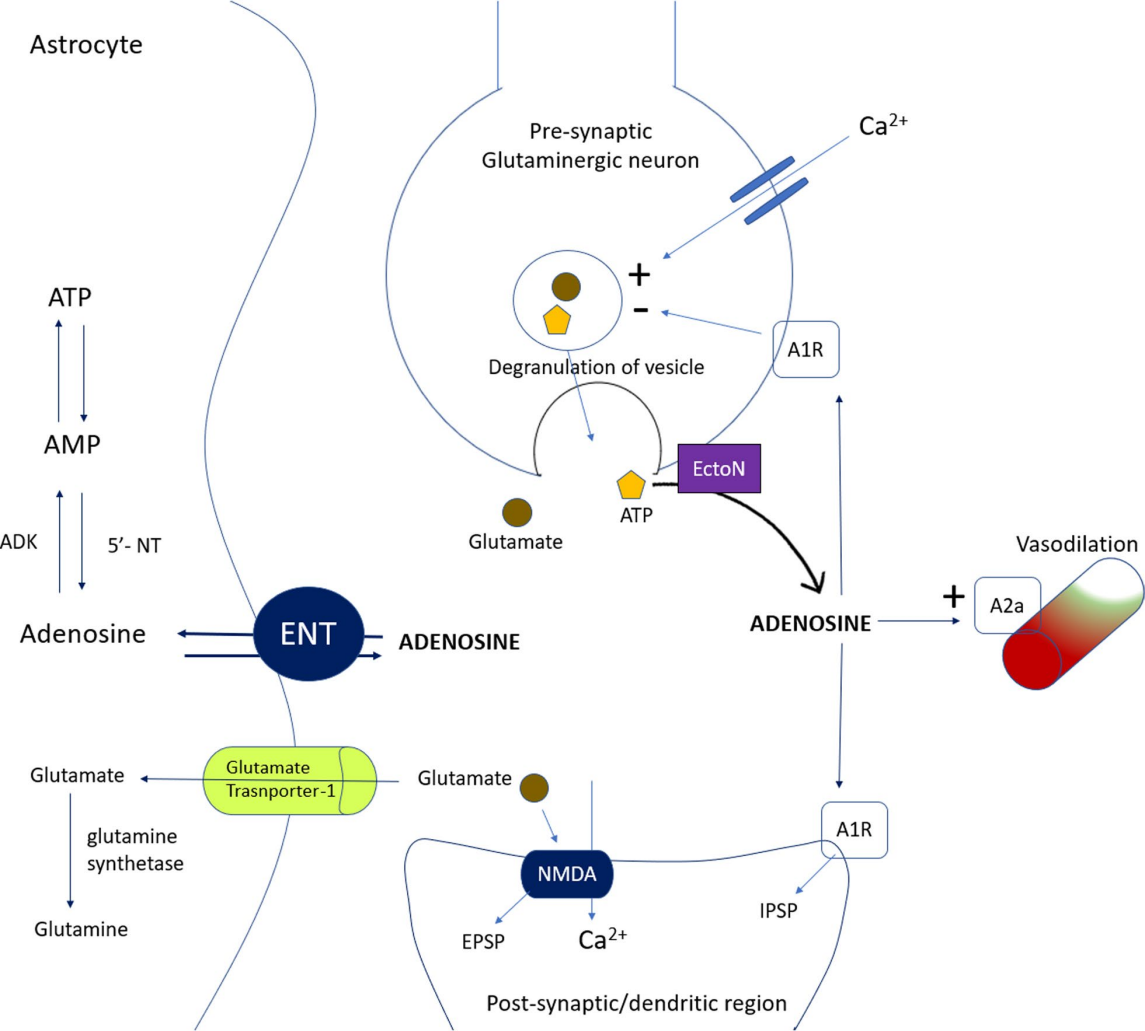
Interaction of adenosine with its receptor in astrocytes and blood vessels

As shown in Fig. 2, ATP (Adenosine Triphosphate) breaks down to AMP (Adenosine Monophosphate) and then to adenosine by CD-39 and CD-73, respectively. Adenosine so formed either binds to its receptor and exerts it physiological/pathological action or is converted to inosine using Adenosine-de-aminase (ADA). Adenosine migrates inside the cell using ENT (Equilibrative nucleoside transporter). Inside the cell, adenosine is serially phosphorylated to form ATP which migrates outside the cell using connexin (a transmembrane protein in cell membrane) [39, 40] (Fig. 3).

Figure 3 concerns the role of glial cells in controlling glutaminergic neuronal excitation and modulating neuronal blood flow, which are mediated by adenosine. Astrocytes are star-shaped cells (glial cells) found in brain, with typical neuron-to-glial cell ratio of 1:1. Main function of astrocytes is to clear synaptic neurotransmitter such as GABA and glutamate. It also regulates blood flow on neuronal activation. ATP is stored in presynaptic vesicles with glutamine. They both are released, concurrently, by glutaminergic neurons in response to action potential. As a matter of fact, adenosine appears in synaptic cleft either as metabolic product of adenosine triphosphate (attributed to EctoN or ectonucleotidases) or it is released by astrocytes itself (using ENT or Equilibrative Nucleoside Transporter), in response to ischemia. Adenosine can bind to both pre- and post-synaptic A1R, where it can reduce firing in presynaptic glutaminergic neuron and reduce responsiveness to glutamine in post-synaptic neuron. This property is being explored for developing new anti-epileptic agent. Adenosine can also bind to A2R and can cause vasodilation in response to ischemia and surged neural perfusion needs in response to neuronal activation. This property is being explored to devise pharmacological agents, which can control neurodegenerative disease, here IPSP: Inhibitory Post-Synaptic Potential and EPSP: Excitatory Post-Synaptic Potential [41–43].

#### Adenosine as anti-inflammatory agent

**Fig. 4:**
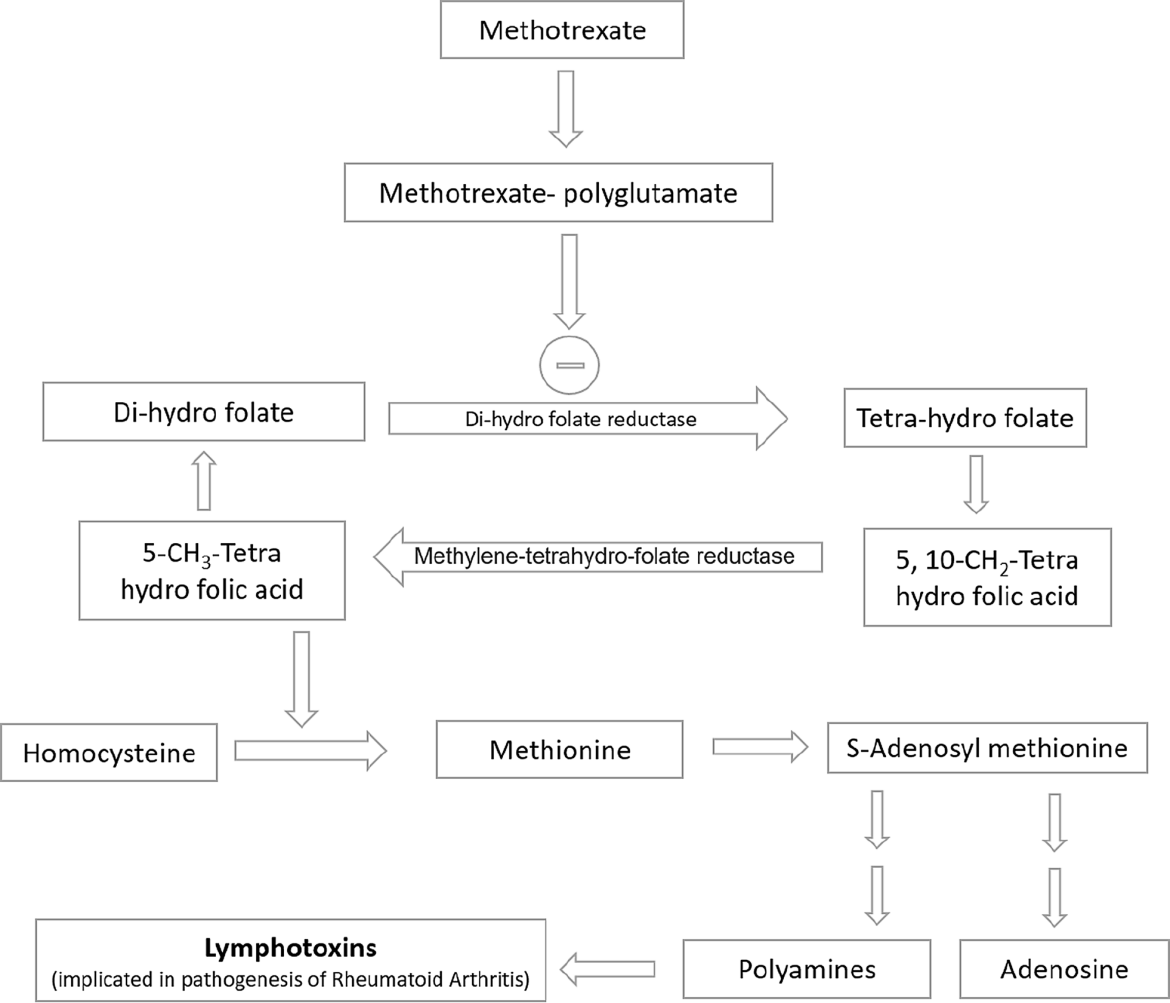
Adenosine mediated inhibition of lymphotoxin and polyamine formation by methotrexate

By activating A2(A) receptors, adenosine shifts the macrophage function from inflammatory factors production (TNF-alpha and IL-10) to anti-inflammatory factor production (VEGF and IL-10). During inflammation, neutrophils adhere to vascular endothelium and then migrate to site of inflammation under influence of chemicals (a phenomena known as chemotaxis or chemo kinesis). After this, they attack pathogens by phagocytosis and by generating reactive oxygen species (ROS)/oxygen radicals. Adenosine by interacting with A2A receptor can potentially retard the above-mentioned events thereby mitigating the inflammation. Adenosine derivative, S-adenosyl methionine, has been indicated as one of the mediators in therapeutic cascade which is modulated methotrexate especially when used in the treatment of rheumatoid arthritis disease. This further justifies the potential that adenosine and its congeners may have in treatment of inflammatory diseases [44–51] (Fig. 4).

As depicted in Fig. 4, first, methotrexate polyglutamate is formed from methotrexate. This event is followed by inhibition of enzyme dihydro-folate reductase. As the cascade progresses, the level of 5-methyl tetrahydro-folic acid decreases leading to reduced conversion of homocysteine to S-adenosyl methionine. This ultimately reduces the levels of lymphotoxin (a mediator indicated in rheumatoid arthritis disease) [52].

#### Adenosine in microbial pathogenesis

In order to survive inside the host by evading its immune system, a plethora of microbes utilize adenosine. This is prominent especially in the case of certain gram-positive microorganisms like *Enterococcus faecalis, Bacillus anthracis, Staphylococcus aureus and Staphylococcus epidermidis.* These microorganisms utilize an extracellular 5’-nucleotidase enzyme called adenosine synthase-A (AdsA) and with its help, these microbes convert adenosine monophosphate (AMP) to Adenosine. It is suggested that adenosine so formed (after binding to A2A receptor) leads to inactivation of multiple protective responses which arises on the part of host’s immune system which includes (but is not limited to) delayed activation of neutrophil and/or macrophages, arrested degranulation of neutrophils, suppression of diapedesis, arrested synthesis of TNF- α and Interleukin-12 in white blood cells [53–59].

#### Adenosine as pain reliever

Recent advances in research have attracted the attention of scientists towards adenosine and its agonist as potential antinociceptive agent. Adenosine A1 receptors are expressed in pain sensitive (nociceptive) neurons of spinal cord which are targets of such drugs. But because of short-lived lower back pain and head ache (as common side effects) along with limited clinical efficacy, adenosine and adenosine receptor agonists have not gain any edge over conventional analgesics clinically [60].

#### Adenosine in penile erection

Adenosine is also associated in erection of penis. This is achieved by relaxing a spongy tissue present in penis known as corpus cavernosum. It is worth to note that erectile dysfunction (ED) is attributed to compromised adenosine signalling. On the contrary, excess of adenosine activity in penis is often associated with a prolonged and painful erection of penis, a condition known as priapism [61–65].

#### Adenosine receptor agonist in medical practice

A drug named Regadenoson (A2 receptor agonist) has been approved for clinical use by USFDA & EMA (in OCT-2008 USFDA and SEP-2010 by EMA). This drug is used for testing functioning of myocardium (myocardial function testing) because of its ability to cause hyperaemia. Regadenoson has also been approved for myocardial perfusion imaging. Adenosine is also used as an anti-arrhythmic drug. It has been found as life-saving drug when cardioversion is tried in the case of paroxysmal supraventricular tachycardia (PSVT). Adenoject® and Adenocor® are two preparations available in Indian markets for such purpose (3 mg adenosine base per ml in 2 ml & 10 ml ampoule) [66–73].

#### Adenosine as antiviral agent

Two congeners of adenosine namely carbocyclic 3-deazaadenosine (C-c3Ado) and carbocyclic 3-deazaadenosine (C-c3Ado) have got attention as antiviral agents. These agents have established efficacy in retarding growth of certain viruses such as rotavirus, vesicular stomatitis virus, rotavirus and reovirus. Adding to in this series, 7-deazaadenosine (tubercidin) and 3-deazaadenosine are also two of adenosine analogues which have wide spectrum antiviral activity. It is worth mentioning here that activation of A2A receptors is implicated in antiviral activity of these agents. Another adenosine nucleoside analogue in this series is BCX-4430 which, after phosphorylation, mimics ATP. This agent is converted to its corresponding triphosphate by cellular kinases. After phosphorylation, this agent gets incorporated in growing RNA chain leading to premature termination of replication process. BCX-4430 was found to have promising in vitro efficacy against SARS-CoV, Ebola virus and MERS-CoV. Its phase 1 clinical trial is in progress [74].

#### Adenosine in cancer immunotherapy

Adenosine has also been implicated in the pathogenesis of cancer. A tumour cell expresses very high levels of CD-73 and CD-39 as compared to normal cell. This leads to high levels of adenosine in tumour microenvironment. Adenosine suppresses the immune system in tumour microenvironment providing tumour an immunity against being attacked by immune cells. This dampening of immune system by adenosine is attributed to A2A receptors expressed on cells of immune system. In future A2A receptor and A2B receptor antagonists are expected to widen new horizons in immunotherapy of cancer. CPI-444 is one of the A2A receptor antagonist molecules which is being studied for the treatment of RCC (Renal Cell Carcinoma) [75, 76].

#### Adenosine as therapeutic agent: recent advances and prospect

In one of the studies, adenosine amine congener (ADAC) has been found to be a promising agent in treatment of Huntington's disease (a neurodegenerative disorder characterized by challenged cognitive and motor functions) [77, 78].

In another research A2A receptor antagonist has been found to be a promising protective agent for 3-nitropropionic acid (3NP, a mitochondrial toxin)-induced striatal neurodegeneration [79, 80]. Adenosine A2A receptor antagonists have also been found to be prospective agents for treatment of Alzheimer’s disease (AD). One such agent is MSX-3, a selective and potent A2A receptor antagonist. In this study, APPsw/PS1dE9 mice (3–9 months of age) were treated for 6 months with MSX-3. This treatment significantly increased Aβ1-40 cortical levels while reduced the cortical levels of Aβ1-42, concluding that A2A receptor antagonist was found to have a promising therapeutic potential for treatment of AD. A selective A1 adenosine receptor agonist, adenosine amine congener (ADAC), has been indicated to have significantly reduced noise-induced hearing loss in Wistar rats (in a time and dose dependent manner). This suggests that adenosine and its congeners can be studied and developed further to make otic preparations as well [81].

An adenosine congener, WS0701 {(2R, 3S, 4R, 5R)-3, 4-dihydroxy-5-[6-[(4-hydroxy-3-methoxybenzyl) amino]-9H-purin-9-yl] tetrahydrofuran-2-yl} methyl decanoate (which is an N6-substituted adenosine derivative), has remarkably prolonged non-rapid-eye movement sleep in mice. This study proved that adenosine and its congeners have potential of being used as anti-anxiety and hypnotic agent [82]. This agent also has markedly increased the Bcl-2/Bax ratio in the hippocampus of mice brains. It also has significantly reduced the stress-induced apoptosis of hippocampal neurons in mice. This study has demonstrated adenosine and its congener as promising agent in treatment of post-traumatic stress disorder [83, 84].

Another adenosine congener WS090501 (binds to adenosine A1 receptor) has been found to significantly reduce spontaneous locomotion and increase non-rapid-eye movement sleep in mice. It also prolonged latency of pentylenetetrazole-induced convulsions. All these activities confirm its sedative, hypnotic and anticonvulsive nature. WS090501 may be utilized in future in treatment of seizures and convulsions [85, 86]. Adenosine receptors can control glutamatergic and dopaminergic neurons and they have a neuro-modulatory role which can be utilized in treating schizophrenia [87, 88]. Adenosine A2 receptor and dopaminergic D2 receptors interact. They have intra-membrane antagonistic interaction which may open up new perspective in treatment of schizophrenia with adenosine congeners [89–93]. 5′-Cl-5′-deoxy-ENBA, a selective adenosine A1 receptor agonist inhibited hyperlocomotion produced by amphetamine in rodents which suggest that 5′-Cl-5′-deoxy-ENBA has antipsychotic action [94]. Adenosine has also been attributed in inducing hypometabolic and hypothermic state in some animals during torpor [95].

## Conclusion

Although an excellent advancement has been made in understanding the role of adenosine in physiology and pathogenesis, yet the number of formulations of adenosine and its congeners in markets are limited. More advancement has been warranted to achieve a breakthrough in treatment of inflammation/inflammatory disease, erectile dysfunction, neurodegenerative disease and infections using adenosine and its congeners.

## Data Availability

All the information in the manuscript has been referred from the included references and is available upon request from the corresponding author.

## Abbreviations

ATP: Adenosine triphosphate
DNA: Deoxy-ribonucleic acid
RNA: Ribonucleic acid
SAM: S-adenosyl-L-methionine
GPCR: G protein-coupled receptor
PSVT: Paroxysmal supraventricular tachycardia
ENTs: Equilibrative nucleoside transporters
SAHH: S-adenosyl-homocysteine hydrolase
ADP: Adenosine diphosphate
CD-73: Cluster of differentiation-73
AdoHcy: S-(5′-Adenosyl)-L-homocysteine
EctoN: Ectonucleotidase
cAMP: Adenosine 3′ 5′-cyclic monophosphate
ED: Erectile dysfunction
ADAC: Adenosine amine congener
3NP: 3-nitropropionic acid
